# Socio-cultural factors associated with knowledge, attitudes and menstrual hygiene practices among Junior High School adolescent girls in the Kpando district of Ghana: A mixed method study

**DOI:** 10.1371/journal.pone.0275583

**Published:** 2022-10-04

**Authors:** Lebene Kpodo, Matilda Aberese-Ako, Wisdom Kudzo Axame, Martin Adjuik, Margaret Gyapong

**Affiliations:** 1 Department of Learning and Evaluation, Pencils of Promise, Ho, Volta Region, Ghana; 2 Institute of Health Research, University of Health and Allied Sciences, Ho, Volta Region, Ghana; 3 School of Public Health, University of Health and Allied Sciences, Ho, Volta Region, Ghana; 4 School of Basic and Biomedical Sciences, University of Health and Allied Sciences, Ho, Volta Region, Ghana; University Medical Center Utrecht, NETHERLANDS

## Abstract

**Background:**

Good menstrual hygiene practice is critical to the health of adolescent girls and women. In Ghanaian public schools, the School Health Education Program which includes menstrual health education has been instituted to equip adolescents with knowledge on menstruation and its related good hygiene practices. However, in most communities, menstruation is scarcely discussed openly due to mostly negative social and religious beliefs about menstruation. In this study, we examined socio-cultural factors associated with knowledge, attitudes and menstrual hygiene practices among Junior High School adolescent girls in the Kpando Municipality of Ghana.

**Materials and methods:**

A mixed method approach was employed with 480 respondents. A survey was conducted among 390 adolescent girls using interviewer-administered questionnaires to collect data on knowledge of menstruation and menstrual hygiene practices. Focus Group Discussions (FGDs) using a discussion guide were conducted among 90 respondents in groups of 9 members. The FGD was used to collect data on socio-cultural beliefs and practices regarding menstruation. Descriptive and inferential statistics and content analysis were used to analyze the quantitative and qualitative data respectively.

**Results:**

Most (80%) of the study participants had good knowledge of menstruation. Also, most (82%) of the participants practiced good menstrual hygiene. Attending a public (AOR = 0.24, 95% CI = 0.12–0.48, p<0.001) and rural (AOR = 0.40, 95% CI = 0.21–0.75, p<0.01) school was significantly associated with reduced odds of practicing good menstrual hygiene. Good knowledge of menstruation was associated with increased odds of good hygiene practices (AOR = 4.31, 95% CI = 2.39–7.90, p<0.001). Qualitative results showed that teachers provided adolescents with more detailed biological information on menstruation than key informants (family members) did at menarche. However, both teachers and family members spoke positively of menstruation to adolescent girls. Social and religious beliefs indicate that menstruation is evil and unclean. Such beliefs influenced community members’ attitudes towards adolescent girls and led to practices such as isolating menstruating girls and limiting their ability to interact and participate in certain community and religious activities.

**Conclusion:**

Despite the prominence of negative social and religious beliefs about menstruation, good menstrual hygiene practice was high among study participants. Knowledge of menstruation; place of residents; and type of school were the major factors associated with good menstrual hygiene practice. It is therefore, necessary to intensify the School Health Education Program in both rural and urban public and intensively involve private schools as well to ensure equal access to accurate information on menstruation and good menstrual hygiene practices among adolescent girls.

## Introduction

Menstruation is a physiological phenomenon experienced by adolescent girls and pre-menopausal women. It is characterized by the monthly flow of blood from the uterus through the vagina from puberty to menopause, which is a result of the shedding of the lining of the uterus [1]. It is caused by hormones [1] and the normal menstrual cycle is between twenty-one to thirty-five days [2]. This physiological occurrence is the same for girls and women globally but cultural differences account for contextual meanings ascribed to menstruation and the provisions made for the attainment of Menstrual Health (MH) [3,4]. MH has been defined by Hennegan et al. (2021) as a state of complete physical, mental, and social well-being and not merely the absence of disease or infirmity, in relation to the menstrual cycle [5].

Globally, at least an estimated 500 million adolescent girls and women are unable to attain MH due to their inability to easily access basic menstrual hygiene management essentials [6]. Menstrual Hygiene Management (MHM) essentials include accurate information on menstruation and menstrual hygiene, menstrual hygiene materials, and a supportive socio-cultural environment [5,7]. Although in recent times, MH has seen significant recognition in terms of advocacy and programming globally [5], in some parts of the world, especially in low-resource countries, women are still not well-equipped with holistic biological information on menstruation and hence are not able to pass on accurate knowledge on menstruation to adolescent girls [4]. They also shy away from discussing menstrual related topics with adolescent girls therefore, adolescent girls have limited knowledge of menstruation [4]. Studies conducted among adolescent girls in Nigeria and India indicated that the majority of the girls surveyed did not know that menstrual blood comes from the uterus [2,8]. Furthermore, studies conducted in Nigeria and India among adolescent girls, found that majority of them did not know the cause of menstruation [2,8]. This is also the case in Ghana as a study by Nsemo et al. (2020) found that most of the adolescent girls in their study did not know the source of menstrual blood [9]. Boakye-Yiadom et al. (2018) also found that majority of adolescent girls in their study in Ghana had no knowledge of the cause of menstrual flow [10]. Nevertheless, other studies conducted in Ghana indicated that the majority of adolescent girls had knowledge of the normal menstrual cycle [9–12]. Even though some of the previous studies in Ghana suggested that most Ghanaian adolescent girls knew the length of the normal menstrual cycle, they lacked accurate information on the source and cause of menstruation [9–12]. This is disturbing because holistic biological information is critical in MH [5].

Knowledge of menstrual hygiene and the provision of menstrual hygiene materials are also crucial for good menstrual hygiene practice [1,5]. This is because poor menstrual hygiene practice may have profound negative effects on the health of adolescent girls [4,13] and can lead to reproductive tract infections (RTIs). RTIs when left untreated can cause problems with reproduction, such as infertility [14]. Good menstrual hygiene requires the use of a hygienic menstrual absorbent [2,15,16]. Menstruating adolescents and women use menstrual absorbents such as sanitary pads, toilet rolls or paper, reusable cloth, tampons, menstrual cups, and plant fiber pads. Some studies conducted among adolescent girls in India and Ethiopia revealed that sanitary pad was the most used absorbent [14,16]. Similarly, studies in Ghana also reported that sanitary pad was the most used absorbent during menstruation [17,18]. Choice of menstrual absorbents depends on the availability of the absorbent in one’s environment, individual preference, cultural acceptability, and financial ability to afford the absorbent [15]. Additionally, cleaning the genitalia during menstruation with clean water is also critical [7,19] This implies that access to clean water is important in menstrual hygiene management [7,19,20]. In a study in Western Ethiopia, majority of the adolescent girls cleaned their genitalia with clean water [16]. Another study conducted among adolescent girls in Nigeria reported that less than 50% of girls cleaned their genitalia during menstruation [2]. However, in Ghana, majority of the girls cleaned their genitalia with water during menstruation [20].

In low-and-middle-income countries, menstruation is surrounded by taboos, restrictions and misconceptions [13,17]. In India, some menstruating girls were not permitted to engage in any form of religious activities [21]. In some parts of Nepal, women are forced to practice menstrual exile [13]. These restrictions are mostly based on the misconception that menstrual blood is unclean hence menstruating women are also unclean [13,22]. In Ghana, some menstruating women are restricted from religious activities [17]. A study conducted in Ghana revealed that discussion on menstruation was considered a taboo and the misconception that menstruating girls and women are unclean was widespread. These taboos and misconceptions about menstruation have resulted in a culture of silence surrounding menstruation. This makes it difficult for adults to educate adolescent girls on menstruation before menarche [17].

As advocacy for MH has intensified in recent times, the Ministry of Education (MoE) in collaboration with the Ministry of Health (MoH) which instituted the School Health Education Program (SHEP) in pre-tertiary public (government-owned) schools in 1992, has inculcated menstrual hygiene management in its program to break the culture of silence on menstruation and related practices and to support students to live healthy reproductive lives. The SHEP had been supported by the United Nations Children’s Fund (UNICEF) and an international Non-Governmental Organisation (NGO) known as Plan International to intensify MHM education in pre-tertiary schools. The pre-tertiary schools include the Junior High School (JHS) level which is a three-year educational program made up of three grades namely JHS 1, JHS 2, and JHS 3. Majority of the JHS students are between the ages of 12 to 16 years old [23] hence interventions such as the SHEP are critical at this level. Under the SHEP, the Ghana Education Service (GES) and Ghana Health Service (GHS) carry out intensive education on reproductive health topics such as menstruation and good menstrual hygiene practices [24,25] to ensure that adolescents receive ample knowledge and are well prepared for practicing good menstrual hygiene [26]. Additionally, UNICEF and Plan International have supported the SHEP program through training of School-based Health Coordinators (SBHC) on MH and have provided hygiene facilities such as toilets and handwashing facilities to the schools [24]. Also, the program is regularly monitored by district SHEP coordinators. Furthermore, the JHS curriculum emphasizes education on adolescents’ reproductive health which includes MH hence it is taught in private (non-government owned) schools as well [24].

Some of the recent studies in Ghana suggested high knowledge of menstruation and the prevalence of good menstrual hygiene practices among Junior High School adolescent girls [9,18]. However, Mohammed and Larsen-Reindorf (2020), reported poor knowledge of menstrual hygiene among Junior High School adolescent girls in Ghana [17]. Boakye-Yiadom et al. (2018) on the other hand, reported that knowledge of menstruation did not translate into good menstrual hygiene practices, as merely one out of three study participants who had knowledge of menstruation practiced good menstrual hygiene. Nevertheless, all the studies in Ghana reported that the sources of knowledge on menstruation were mostly from mothers and relatives and adolescent girls whose mothers had obtained basic formal education had more knowledge and practiced good menstrual hygiene [9,10,17,27]. Only Nsemo et al. (2020) reported that 37% of adolescent girls in their study had obtained information on menstrual hygiene from their teachers [9]. Additionally, despite the implementation of the SHEP, some recent studies reported that menstruation still serves as a social and cultural barrier to adolescent girls due to traditional and religious beliefs that menstrual blood is dirty, hence, menstruating women are perceived as unclean [18,28,29]. Girls, thus, suffer from discrimination and stigma which affect their ability to enjoy fulfilling and healthy lives [18]. These findings are a matter of concern for the country as well as for institutions such as the GES, GHS, and their international partners because they have been providing adolescent girls in public schools with knowledge and training on good menstrual hygiene practices through the SHEP. For Ghana to achieve the set target of sustainable development goal **3.7: “***By 2030*, *ensure universal access to sexual and reproductive health-care services*, *including for family planning*, *information and education*, *and the integration of reproductive health into national strategies and programmes”* [30] and to ensure that the goal of article 5(a) of the Convention on the Elimination of All Forms of Discrimination against Women (CEDAW): “*To modify the social and cultural patterns of conduct of men and women*, *with a view to achieving the elimination of prejudices and customary and all other practices which are based on the idea of the inferiority or the superiority of either of the sexes or stereotyped roles for men and women*” [31] are realized, there is the need to carry out more studies to understand the experiences of girls to inform policy. Consequently, this study examined socio-cultural factors associated with knowledge, attitudes, and menstrual hygiene practices among Junior High School adolescent girls in the Kpando Municipality of Ghana.

## Materials and methods

### Study design

The study was cross-sectional and employed a mixed-method which was parallel in approach to collect data among in-school JHS adolescent girls between the ages of 10 to 19 years within the Kpando Municipality, from February to May 2018. The main inclusion criterion was girls between 10 to 19 years, who menstruate or have experienced menarche. Girls of this age category who had not attained menarche were excluded from the study. This was because only those who had attained menarche could provide accurate information on the main variable of interest, which is menstrual hygiene practice. A mixed-method was employed for the study because the quantitative method could not be used to adequately explore all aspects of the study especially, socio-cultural beliefs and practices on menstruation. Therefore, a survey was used to collect data on knowledge of menstruation and menstrual hygiene practices while the qualitative method employed Focus Group Discussions (FGDs) to collect data on socio-cultural beliefs and practices regarding menstruation. FGD was used for the study because it allowed respondents to share their intimate experiences on menstruation, which helped the researchers gain insight into various dimensions of the phenomena of interest [32].

### Study setting

The Kpando Municipality is one of the 25 Districts in the Volta Region of Ghana, with a total population of 53,736 and a growth rate of 2.5%. Females constitute 52% (27,832) and males form 48% (25,904). Adolescent girls constitute 30% of the female population of the Municipality [33]. About 32% of the adult population are engaged in agriculture, 24% in service and sales, 22% in craft and related trade, and 11% are engaged in other sectors. The Municipality is dominated by the Ewe ethnic group. Majority (89%) of the population in the Municipality are Christians, 6% are Muslims and 5% belong to the African traditional religion and other religions. The literacy rate of the Municipality is 86% [34]. The Municipality has 42 JHSs comprising 31 public/government-owned schools and 11 private/non-government-owned JHSs. Since the institution of the SHEP program in Ghana, it has been rolled out in pre-tertiary schools in the Kpando Municipality [24].

### Sampling procedures

A total of 390 students were sampled for the survey and 90 were selected for the FGDs.

We used Cochran’s formula [34] to determine the required sample size for this study. Assumptions made during the sample size calculation were P = 31.1% (prevalence of good menstrual hygiene management) based on an earlier study conducted in Yendi by Boakye-Yiadom and colleagues (2020) [15], 95% confidence interval, and a 5% margin of error. This yielded a total sample size of 362. The sample size was upwardly adjusted to 390 to cater for non-response.

A stratified sampling procedure [34] was used to select participating schools. The Ghana Education Service municipal directorate has stratified schools in the Kpando Municipality into five administrative strata called circuits with an average of seven schools in each circuit. A total of ten schools were selected (two each from each of the five administrative circuits) for the study. A two-stage sampling procedure was employed for the selection of the study participants. Firstly, schools were selected as follows: (i) a list of all JHSs in each circuit was obtained from the Municipal education office and was used as the sampling frame; (ii) two schools were sampled from each stratum using the non-replacement lottery method. Seven of the ten selected schools were public schools and three were private schools. Secondly, study participants were selected as follows: (i) using the formulae by Cochran: n/N*390 (where: n = enrollment of girls in a school, N = Total enrollment of girls in the 10 schools, and 390 = the study sample size), the sample size for each school was determined using proportion to sample size; (ii) a list of enrolled adolescent girls in each school was collected from the head teacher and permission was sought from the head teachers to interact with the girls; (iii) The girls on the lists provided by each head teacher were called into a private room and the research team, who were females together with the SBHC interacted with them on common social issues as a way of building rapport between the students and the research team. Thereafter, the girls were called one after the other into a private room by the SBHC and the researchers and were asked whether they had attained menarche. The names of those who indicated that they had not attained menarche were removed from the list; (iv) ‘Yes’ and ‘No’ were written on paper, folded and mixed in a box [34]. The number of papers with ‘Yes’ on them placed in each box was per the sample size calculated for each school and the papers with ‘No’ written on them was per the excess number of the eligible participant when the calculated sample size was deducted from the total number of eligible participants. Those who had attained menarche were asked to pick a paper each from the ballot box. A total of three hundred and ninety picked ‘Yes’.

For the FGDs, 90 female students, 9 each from each of the 10 selected schools, who had attained menarche and volunteered to speak freely on menstruation were included in the FGDs.

The researchers explained the study as well as issues regarding confidentiality, voluntary participation, and the right to withdraw from the study at any point in time to all the prospective study participants. They were subsequently given consent forms (18 years and above) and assent and parental consent forms (those below 18 years) to send home three days before the interview day. All the students returned signed form(s) which was an indication that they and or their parents have consented to participate in the study and this was the last criteria for recruitment into the study.

### Data collection and analysis

#### Quantitative data collection and analysis

The quantitative data was collected using an interviewer-administered questionnaire, which was adapted from other authors [2,10,16], modified and pre-tested by the study team. The pre-test was conducted among adolescent girls with similar characteristics as the targeted study population in two selected schools and based on the outcome, the questionnaire was edited and finalized. The questionnaire was written in the English Language, however, it was translated into the local language of the respondents (Ewe is the language spoken in the study area and English is the official language of Ghana), and digitized using CSPro software and transferred onto android devices. The questionnaire was divided into three sections namely; Section A: Socio-demographic characteristics of the respondents; Section B: Knowledge of menstruation and Section C: Menstrual hygiene practices. The questionnaire was administered using either English or Ewe based on the preference of the respondents in a private room through face-to-face interviews with each participant by six experienced and well-trained female researchers who had Bachelor’s degree in Public Health and spoke English and Ewe fluently. The questionnaires were administered by the interviewers because not all the respondents were fluent readers.

Data were transferred to Stata Version 14.1, cleaned and validated to ensure quality before it was analyzed. Categorical variables were presented using frequencies and percentages. A scoring system similar to that of Fehintola et al. (2017); Boakye-Yiadom et al. (2018) and Upashe et al. (2015) was adapted and used to assess knowledge of menstruation and menstrual hygiene practices [2,10,16]. A score of 1 was assigned to correct responses and 0 was assigned to incorrect responses. Regarding knowledge on menstruation, five variables were considered; cause of menstruation, source of menstrual blood, the implication of onset of menarche, normal menstrual cycle, and the particular sex that experiences menstruation. The correct responses which were assigned 1 include; hormones as the cause of menstruation; vagina or uterus as the source of menstrual blood; entry into adulthood as an implication of menarche; 21–35 days as normal menstrual cycle; and females as the sex which experiences menstruation. The highest possible score was thus five (5) and the lowest was zero (0). A total score of three (3) and above which was above the median was classified as good knowledge and a total score of less than three (3) was considered poor knowledge of menstruation. A dichotomous variable called knowledge on menstruation was then developed out of the knowledge variables. Good knowledge was coded as 1 while poor knowledge was coded as 0.

Regarding menstrual hygiene practice, three (3) variables were taken into consideration. These included the type of absorbent used; genitalia cleaning during menstruation; and the material used for cleaning the genitalia. The correct responses which were assigned 1 included; sanitary pad, toilet roll, clean cloth as type of absorbent used; cleaning of genitalia during menstruation, and use of at least clean water as genitalia cleaning resource or material. The highest possible score was thus three (3) and the lowest was zero (0). A total score of two (2) and above which was above the median was classified as good menstrual hygiene and a total score of less than two (2) was considered poor menstrual hygiene practice. A dichotomous variable called hygiene practice was then developed out of the practice variables. Good practice was coded as 1 while poor practice was coded as 0.

Multivariable logistic regression was used to determine the strength of association. In the logistic regression model, good hygienic practice was coded 1 and poor hygienic practice was coded 0. The principal dependent variable was hygienic practice and the outcome of interest was good hygiene practices. Strengths of association between the independent variables and good hygiene practices were determined using the crude odds ratio (Model 1). Variables with a p-value of <0.05 in Model 1 were considered for inclusion in the multiple logistic regression analysis (Model 2). However, although one variable thus, location was not significant in Model 1, it was included in Model 2 because it has been widely reported to be a major factor that influences MHM [35–37]. To test for goodness of fit of Model 2, we used the likelihood ratio test to examine the likelihood of data under the full model as against the likelihood of the data under a model with reduced number of independent variables. We obtained a p-value for the overall model to be less than 0.05. Thus, we concluded the model was good.

#### Qualitative data collection and analysis

An FGD guide was used to collect the qualitative data. It was written in English and translated into Ewe. The guide was pre-tested among adolescent girls in the two selected schools where the questionnaire was pretested. Based on the outcome of the pre-test, the guide was revised and finalized for the actual study. Each FGD was made up of nine participants. At each discussion there was a moderator, note taker and observer (32), all of whom were female researchers with Bachelor’s Degree in Public Health. All discussions were held in either English or Ewe per the preference of the respondents and were recorded using a digital recorder. The audio recordings from the FGDs were transcribed verbatim after each session by the note taker and the transcripts were verified by the moderator.

All transcribed notes were typed in Microsoft Word 2016 and analyzed using content analysis. Frequently used words by participants were identified and coded manually after a thorough reading of transcripts. Statements containing the same or similar words were coded and categorized into themes for interpretation and further analysis [32].

### Ethical statement

Ethical approval with reference number UHAS-REC A.6 [17] 17–18, was obtained from the University of Health and Allied Sciences’ Research Ethics Committee (UHAS-REC). Additionally, approval was sought from the Kpando Municipal Education Service before the commencement of data collection. Before inclusion into the study, written individual informed consent was obtained from study participants who were up to 18 years old, while parental consent, as well as assent, were obtained from parents and students below 18 years. All the forms were given to the prospective respondents three days before the interview date to allow them ample time for deliberation to take part in the study or not.

## Results

### Socio-demographic characteristics of the respondents

Majority 306 (78%), of survey participants were between the ages of 14–16 years old (Table 1). Most of them 307 (79%), experienced menarche between 12 to 15 years. Respondents in JHS 1 were 31%, JHS 2 31%, and JHS 3 38%. Majority of the students were Christians 365 (94%) and belonged to the Ewe ethnic group 333 (85%). About half the respondents 194 (50%) lived with their mothers while 24 (6%) lived with their fathers, and 49 (13%) lived with both mother and father.

**Table 1:** Socio-demographic characteristics of the respondents.

Variables	Survey		FGD	
	Frequency (N = 390)	Percentage (%)	Frequency (N = 90)	Percentage (%)
**Age of respondents**				
11–13	36	9.2	18	20.0
14–16	306	78.5	65	72.2
17–19	48	12.3	7	7.8
**Age at menarche**				
<12	24	6.2	26	28.9
12–15	307	78.7	67	67.8
>15	59	15.1	7	3.3
**Class of respondents**				
JHS 1	121	31.0	30	33.3
JHS 2	120	30.8	30	33.3
JHS 3	149	38.2	30	33.3
**Religion**				
Christianity	365	93.6	87	96.7
Islam	25	6.4	3	3.3
**Ethnicity**				
Ewe	333	85.4	77	85.6
Akan	22	5.6	1	1.1
Hausa	24	6.2	12	13.3
Others	11	2.8	0	0.0
**Person lives with**				
Father only	24	6.2	1	1.0
Mother only	194	49.7	32	35.6
Both mother and father	49	12.6	25	27.8
Other relative(s)	123	31.5	32	35.6
**Type of school**				
Public	322	82.6	63	70.0
Private	68	17.4	27	30.0
**Location of school**				
Rural	138	35.4	36	40.0
Urban	252	64.6	54	60.0

For the FGDs, more than half, 65(72%) of the students were between the ages of 14–16 years whilst minority 7(8%) were aged 17-19years (Table 1). An equal number 30 (33%) of students each were in JHS 1, JHS 2 and JHS 3 respectively. Majority, 87(97%) of the students were Christians and 3(3%) were Muslims. More than half, 63(70%) of the students attended public school and 27(30%) attended private school.

### Knowledge on menstruation

Majority 236 (61%) of the respondents correctly indicated that menstruation is caused by hormones. Only 104 (27%) of respondents stated that menstrual blood comes from the womb or uterus, while nearly half of respondents 181(46%), also indicated that it comes from the vagina. Less than half of respondents 187 (48%) appropriately indicated that menarche implied entry into womanhood or the capability of conceiving. Overall, 80% of the respondents had good knowledge on menstruation (Table 2).

**Table 2:** Knowledge of menstruation.

Variables	Frequency	Percentage (%)
**Cause of menstruation**		
Hormones	236	60.5
Disease	13	3.3
Curse from the gods	31	8.0
Don’t know	93	23.8
Food we eat	17	4.4
**Where does menstrual blood come from**		
The womb/uterus	104	26.7
Abdomen	67	17.8
Bladder	6	1.5
Vagina	181	46.4
Stomach/Body	32	8.2
**What does onset of menarche imply?**		
Capable of conceiving/Entry into womanhood	187	48.0
Can start having sexual intercourse	95	24.4
Ready for marriage	61	15.6
Don’t know	25	6.4
Other reasons	22	5.6
**Normal Menstrual cycle**		
< 21 days	75	19.2
21–35 days	176	45.1
> 35 days	12	3.1
Don’t know	127	32.6
**Do boys menstruate**		
No	384	98.5
Yes	6	1.5
**Knowledge**		
Poor knowledge	77	19.7
Good knowledge	313	80.3

The qualitative findings revealed that most of the respondents had good knowledge of menstruation. Below are excerpts from some FGD participants.

*“When you menstruate*, *it means you have reached womanhood*. *Menstruation happens if the egg comes and there is no sperm*, *it breaks and comes out because we cannot keep bad things in our abdomen*.*”* (Participant, FGD02, Public school)

*"It’s the monthly discharge of unwanted blood particles which is as a result of the breakdown of the surface wall of the uterus in a woman*. *The blood comes from the womb*. *When the egg develops and needs sperm to fertilize it and the sperm doesn’t fertilize it*, *it comes out as blood through the vagina*.*"* (Participant, FGD08, Private school)

*“It’s the monthly discharge of blood from the vagina… which is as a result of unfertilized egg… If the egg wants sperm but did not get it*, *the lining of the womb turns into blood and comes out*. *That is what causes the menstruation*.*”* (Participant, FGD09, Private school)

Although, most of the FGD participants had good knowledge of menstruation, there were diverse sources of such information. Teachers and family members educated the adolescent girls on menstruation. Both sources spoke of menstruation positively however, teachers provided more detailed biological information about menstruation than their family members.

Almost all the FGD respondents reported that their teachers taught them Adolescent Reproductive Health topics including menstruation in school. Below are some of their responses.

*“We were taught in school that menstruation begins when we are in adolescence*. *We were taught that once we start menstruation*, *we need to be very careful with boys so that we don’t have sex which may lead to unwanted pregnancy*. *They also taught us that we have eggs in our body and when the eggs do not get any sperm to fuse them*, *they turn into blood and come out… Menstruation is natural and happens at a stage in life*. *When you reach a stage as a girl*, *you have to menstruate”* (Participant, FGD08, Private school)

*“They teach us adolescent reproductive health*. *They teach us how to take care of ourselves to prevent body odor and getting diseases or any other thing*. *They also teach us about menstruation*. *They said it is the discharge of unwanted blood from the body*.*”* (Participant, FGD09, Private school)

*“Madam said when you are menstruating and you did not keep yourself clean*, *you will be smelling*. *She also taught us that*, *when a female reaches a stage she will menstruate and when we are menstruating*, *we should come to school with a pad or what we use so that when it gets to a time that we need to change it then we do that*. *She also told us to use soap and water to wash our hands and throw the water away after changing our pads*.*”* (Participant, FGD04, Public school)

*“They teach us about menstruation and personal hygiene in school*. *They said menstruation means the person has reached womanhood*. *It also means that if you are menstruating you can become pregnant any time you have sex with a man*. *They also said if a girl menstruates she has to bathe well*. *If you are menstruating you have to change your pad very often”* (Participant, FGD07, Public school)

Additionally, most of the adolescent girls sought information about menstruation from their family members at menarche. Most of the girls indicated that at menarche, they were informed that menarche is a sign of entry into womanhood hence they should not be scared. However, the information provided to the study participants by family members at menarche did not include ample details on the biological process of menstruation. The following were some of the responses:

*“My auntie told me that now I am a woman…I should not be getting close to boys*, *otherwise I will get pregnant*.*”* (Participant, FGD05, Public School)

*“… my mother told me that it is not any bad thing so I should not be scared…”* (Participant, FGD08, Private School).

*“… first thing she [mother] said was that I am now a woman*, *so anything can happen*. *Thus*, *if I get intimate with a male*, *I will get pregnant*.*”* (Participant, FGD01, Public School).

Although the information the adolescent girls were provided with at menarche was not detailed enough, it did not include myths and misconceptions. Such beliefs were however very common and study participants experienced mostly negative attitudes in interactions with some community members. These beliefs largely emanated from religious and superstitious beliefs.

#### Religious beliefs

Participants in FGDs perceived that menstrual blood was filthy and impure. Therefore, menstruating women were impure and could pollute or contaminate anything that they came into contact with during menstruation. Such actions could even attract the wrath of the gods, especially when the objects were linked to the supernatural as revealed by the following excerpts:

*"*…*you cannot go to the riverside if you are menstruating*, *because you can contaminate the water and the gods will get angry with you … When you are menstruating you are impure*, *you are not clean*, *so you do not have to enter the river*.*"* (Participant, FGD07, Public School).

*“In my house*, *my grandfather does not allow us to touch his bucket when you are menstruating*. *When you touch my grandfather’s bucket*, *it will get contaminated*.*”* (Participant, FGD10, Private School).

Shrines, mosques and some churches are believed to be holy places and could be contaminated by menstruating women, who are deemed to be unholy during menstruation. Menstruating women are therefore prohibited from coming close to such places and in some instances, they have to sit outside. This perception even reflected in some homes, as menstruating women were excluded from certain spaces. The following responses attest to this experience:

*“My church*, *if you are menstruating*, *you would have to stay outside … to God if you are menstruating you are not clean*. *If a normal human being does not allow his wife to enter a particular place*, *how much more God*?*”* (Participant, FGD09, Public School).

*“I live with my grandfather in his shrine and if you are living in a shrine and you are menstruating*, *you would have to leave to another house… Anytime I am in my menses I leave to another house*.*”* (Participant. FGD02, Private School).

#### Superstitious beliefs

Respondents were of the view that menstrual blood was powerful and thus menstruating women possess supernatural powers that can be used positively and negatively.

Positive uses of menstruating women’s spiritual power include the belief that a menstruating woman has the supernatural ability to render spells and charms powerless. It is believed that consumption of food prepared by a menstruating woman or a person merely sharing objects such as buckets with a menstruating woman could render spells and charms impotent and thus freeing the person from such attacks.

*“Some of our fathers have dangerous spells*, *so when we cook for them whilst in our menses*, *the spell becomes deactivated*.” (Participant, FGD05, Public School)

*“If someone is under a spell or a charm and eats food prepared by a menstruating woman*, *the spell or charm becomes powerless*.*”* (Participant. FGD04, Public School)

Menstruating women are believed to have the supernatural ability to bring bad luck to anyone who comes into contact with them. Consequently, some community members frown upon menstruating girls and women coming close to them, as they believe it could destroy one’s economic fortunes and other opportunities. Study participants shared their experiences as follows:

*"I was about to get close to somebody and he asked me whether I was clean [not menstruating] and I said yes*. *So*, *we forgot about that*. *Another time I asked him why he asked me that question*. *He told me that because he is a man and he is a working [businessman]*, *he believes that if a menstruating woman gets close to him*, *it will reduce his luck and he may not make much money*.*"* (Participant, FGD06, Public School).

*“…*. *They say if you are menstruating*, *you have bad luck*. *So*, *if you bath before anybody baths*, *you will bring bad luck to that person*.*”* (Participant, FGD03, Public School).

### Menstrual hygiene practice

Majority 369 (95%), of the respondents, used sanitary pads during their last menstruation (Table 3). Almost half 191 (49%), of the respondents changed their sanitary pads three times a day. Most 330 (85%), of them cleaned their genitalia and majority 321 (99%), used clean water to do so. Overall, 321 (82.3%) of the respondents practiced good menstrual hygiene.

**Table 3:** Menstrual hygiene practice.

Variable	Frequency	Percentage (%)
**Type of absorbent used**		
Sanitary pad	369	94.6
Toilet roll	9	2.3
Clean cloth	12	3.1
**Number of times absorbent was changed per day**		
1	2	0.5
2	182	46.7
3	191	49.0
4	11	2.8
5	4	1.0
**Genitalia cleaning**		
No	60	15.4
Yes	330	84.6
**Genitalia cleaning material**		
Unclean water	3	0.9
Clean water	327	99.1
**Place of disposing absorbent**		
Open field	2	0.5
Burn	68	18.0
Dustbin	144	38.1
KVI P Toilet	164	43.4
**Absorbent wrapper**		
Paper	342	90.5
No wrapper	36	9.5
**Material used in Washing used absorbent (Cloth)**		
Water and Soap	10	83.3
Water only	2	16.7
**Place for drying cloth**		
Enclosed area without sunlight	6	50.0
Open place with sunlight	6	50
**Menstrual hygiene practice**		
Good	321	82.3
Poor	69	17.7

Respondents indicated in FGDs that at menarche, they were encouraged to practice good hygiene during menstruation and sanitary pad was the main absorbent, which was introduced to them. They were also encouraged to practice good personal hygiene during menstrual period.

“*My auntie told me that now I am a woman*, *so I should bath twice a day…*” (Participant, FGD10, Private School)

*“… my mother told me that it is not any bad thing*, *so I should not be scared … she gave me sanitary pad and taught me how to wear it*.*”* (Participant, FGD07, Public school)

*“… first thing she [mother] said was that I am now a woman*… *She [mother] added that at this stage*, *I have to ensure good personal hygiene*, *when I am menstruating and also change my sanitary pads frequently*, *otherwise I will have bad odour*.*”* (Participant, FGD03, Public school)

### Factors associated with good menstrual hygiene practice

Respondents in private schools were less likely to practice good menstrual hygiene (Table 4) compared with those in public schools (AOR = 0.24, 95% CI = 0.12–0.48, p<0.001). Attending a rural school was also associated with reduced odds of good menstrual hygiene practices (AOR = 0.40, 95% CI = 0.21–0.75, p = 0.01). Students who had good knowledge of menstruation were 4.31 times more likely to practice good menstrual hygiene compared to those with poor knowledge (AOR = 4.31, 95% CI = 2.39–7.90, p = 0.001).

**Table 4:** Factors influencing good menstrual hygiene practice.

Variables	Poor hygiene practices n = 69	Good hygiene practices n = 321	Model I COR (95% Cl)	Model 2 AOR (95% Cl)
**Age of respondent (years)**				
11–13	2(2.9)	34(10.6)	1	
14–17	61(88.4)	245(76.3)	0.24(0.06–1.01)	
18–19	6(8.7)	42(13.1)	0.41(0.08–2.17)	
**Age at menarche(years)**				
<12	6(8.7)	18(5.6)	1	
12–14	53(76.8)	254(79.1)	1.60(0.61–4.21)	
15+	10(14.5)	49(15.3)	1.63(0.52–5.14)	
**Type of school**				
Public	48(69.6)	274(85.4)	1	1
Private	21(30.4)	47(14.6)	0.39(0.21–0.71) ***	0.24(0.12–0.48) ***
**Location of school**				
Urban	39(56.5)	213(66.4)	1	1
Rural	30(43.5)	108(33.6)	0.66(0.39–1.12)	0.40(0.21–0.75) **
**Ethnicity**				
Ewe	58(84.1)	275(85.7)	1	1
Akan	2(2.9)	20(6.2)	1.74(0.45–6.67)	1.21(0.31–4.79)
Hausa	8(11.6)	16(5.0)	0.41(0.17–0.99)*	0.40(0.15–1.03)
Other tribes	1(1.5)	10(3.1)	1.49(0.26–8.41)	1.27(0.20–7.92)
**Heard about menstruation before menarche**				
No	43(62.3)	209(65.1)	1	
Yes	26(37.7)	111(34.9)	0.89(0.52–1.51)	
**Knowledge on menstruation**				
Poor knowledge	29(42.0)	48(14.9)	1	1
Good Knowledge	40(58.0)	273(85.1)	4.12(2.34–7.28) ***	4.31(2.39–7.90) ***

*p<0.05

**p<0.01

***p<0.001.

## Discussion

The findings of this mixed-method study revealed that most study participants practiced good menstrual hygiene. Also, the study found that majority of them got some basic information from female relatives and associates on menstruation, however, the information lacked details on how and why menstruation occurs. Nevertheless, attitudes of community members towards menstruation and menstruating females were largely negative. This was influenced by myths and misconceptions about menstruation, which emanated from social and religious beliefs that contributed to menstruating females being restricted from certain spaces and interactions with other community members.

Results from the FGDs and survey revealed that most of the respondents had good knowledge of menstruation. The FGD results reveal that the information provided to the respondents by their teachers was more detailed than the information their close female relatives provided them as it lacked details on menstruation. Similar studies conducted in Nigeria and Ghana revealed that only about half of adolescent girls had good knowledge of menstruation [2,12,17]. The reason for the higher knowledge score in this current study as compared to some studies could be due to the SHEP that is being implemented in public schools coupled with curriculum based MH education in both public and private schools in the Kpando Municipality.

It is important to note that while teachers and family members spoke positively about menstruation to the adolescent girls, myths and misconceptions contributed to negative views about menstruation and mostly negative attitudes of community members toward menstruating females. This is quite worrying, as it could be the reason why the subject is not being thoroughly explored and discussed in depth outside the school system. Additionally, it contributes to adolescent girls not being encouraged to seek the needed support from relatives. Other studies in Kenya, Ghana, Senegal and Nepal have reported that information provided to adolescents on menstruation mostly contains misconceptions, taboos and restrictions [4,38–40].

Although education received on menstruation by the respondents excluded misconceptions about menstruation, findings from the FGDs revealed that religious and superstitious beliefs were prominent. Menstruating women and girls were considered unclean and hence were excluded from some religious and social activities. Some respondents from this study who reported that they live in shrines or belong to certain religious groups were required to stay outside until the period of menstruation had elapsed. Others were restricted from using the same bucket as everyone else, because they were menstruating. In India, a study reported that menstruating girls were not permitted to engage in any form of religious activities [21]. In certain parts of Nepal, menstruating women are required to live apart from everybody else [13]. Restrictions on menstruating girls and women across cultures are disturbing. In Zambia, girls at menarche are expected to seclude themselves from the community [22]. These restrictions translate into stigma, shame and disgrace, which may have a profound effect on the psychological health of women and girls [13].

This study found that sanitary pad was used by most of the respondents and they changed them twice or more daily. This result is in congruence with some other Ghanaian studies in which more than half of girls surveyed used sanitary pads as absorbent during menstruation and also changed the absorbent twice or more daily [41,42]. However, a study in Nigeria reported that cloth was used by most adolescent school girls as absorbent during menstruation and that most of the girls changed the absorbents once a day [2]. The high use of sanitary pads in this current study could be attributed to cultural alterations resulting from modernism, which has favored the use of sanitary pads over the use of traditional materials such as cloth [42]. Also, the qualitative result shows that the respondents were introduced to sanitary pads at menarche, which might have accounted for the high rate of use of sanitary pads.

Our results also revealed that most students cleaned their genitalia during menstrual period. This result is in agreement with a study conducted in Ethiopia among high school girls, which reported that almost all the respondents cleaned their genitalia during menstrual periods [16]. On a contrary, a Nepalese study conducted among adolescent girls reported that only about half of girls cleaned their genitalia during menstruation [40]. The reason for the high rate of cleaning of genitalia in the current study could be as a result of the implementation of the SHEP program and the curriculum-based MH education in Ghanaian schools under which the essence of good hygiene during menstruation is vigorously promoted. Also, information on good personal hygiene practices received at menarche might have accounted for this finding.

We found that the majority of the respondents practiced good menstrual hygiene. A study in India reported similar findings among adolescents in India [43], majority of the respondents practiced good menstrual hygiene. Nonetheless, other studies conducted in Ghana, Ethiopia and Nepal reported that less than half of school girls practiced good hygiene during menstruation [10,16,44]. The finding that majority of the respondents in this current study practiced good menstrual hygiene can be attributed to information they received from their female relatives at menarche. Most of the family members who the girls sought information from at menarche spoke of menstruation positively. A positive approach to education on menstruation reduces anxiety, and fear and encourages good menstrual hygiene practices. Furthermore, the need for maintaining good menstrual hygiene was emphasized and could have accounted for good menstrual hygiene practices by majority of the respondents. Also, most of the respondents had good knowledge of menstruation and that could have contributed to good menstrual hygiene practice among most of the respondents.

This study found that location of school was associated with good menstrual hygiene practices. Students from rural schools were less likely to practice good menstrual hygiene compared to those in urban areas. Similarly, Kuhlmann et al.’s (2017) systematic review study indicated that many school-based studies recorded poorer menstrual hygiene among girls in rural schools compared to those in urban schools [45]. This may be because rural dwellers may not have access to hygiene facilities and may have internalized socio-cultural beliefs that hinder good menstrual hygiene practice. Also, adolescents in urban areas have more exposure to mass media than those in rural areas and exposure to mass media influences knowledge of menstrual hygiene [46]. This could also be a contributing factor to our findings.

The study also found that students who attended private schools were less likely to practice good menstrual hygiene than those who attended public schools. The finding of this study may be because private school students are taught MH as a lesson in the curriculum without any other intervention such as the SHEP. They are not generally included in health programs by the government and NGOs. Most of the health programs are implemented in only public schools and this might have accounted for this finding.

Students in our study who had good knowledge of menstruation were found to be more likely to practice good menstrual hygiene as compared to those with poor knowledge. This compares with the findings of Ameade and Gati (2020) who revealed that respondents in the Northern part of Ghana with good knowledge of menstruation were more likely to practice good menstrual hygiene compared to those with poor knowledge of menstruation [12]. This may be because good knowledge of menstruation reduces misconceptions about menstruation, therefore, paving way for good menstrual hygiene practices. Knowledge of menstruation and good hygiene practice has been recognized in literature as having an essential influence on menstrual hygiene practice just as the present study also found [12,16]. This study, therefore, corroborates the assertion that good knowledge translates into good practice. It is therefore not surprising that a study among adolescent girls in Ethiopia reported poor hygienic practices during menstruation because of poor knowledge of adolescent girls on the subject [47].

Despite the important findings made in the present study, it is worth noting the possible limitations inherent in its conduct. In selecting participants for the focus group discussion, volunteers from each of the three classes were chosen. There is a possibility of bias in the selection. Also, our results were mainly based on responses provided by students on events that occurred in the past (though not too distant). There is therefore, the possibility of recall bias on the part of the respondents. It is however, important to note that these potential limitations do not affect the validity and reliability of our findings in any way.

## Conclusion

Good knowledge of menstruation was prominent among study respondents, but good menstrual hygiene practice was more pronounced among the students. However, the influence of socio-cultural factors such as place of residence, religious and social beliefs as well as myths on menstruation contributed to negative community attitudes towards menstruation and menstruating females in communities. Socio-cultural factors affect girls’ access to certain spaces in communities, confidence and ability to seek crucial information about menstrual hygiene. This makes the need for menstrual education not only an issue for only girls but a crucial matter for the entire society. Nevertheless, the positive approach to menstrual hygiene education used in both the school and household environment could be contributing factors to the prominence of good menstrual hygiene practices among respondents.

Since knowledge of menstruation, type of school attended, and place of residence were associated with good menstrual hygiene practice, it is pertinent to intensify the MH education under the SHEP. This will equip all school-going adolescents with knowledge of MH. As a matter of urgency, the Ministry of Education should ensure that the SHEP Program is extended to private schools to enable female students in such schools to benefit from the program. Additionally, the National Commission for Civic Education should inculcate MH education in their programs in both rural and urban areas to reduce misconceptions about menstruation and to ensure that out-of-school adolescent girls also benefit from the program. Such an initiative will benefit adolescent boys and men as well, who would learn more about menstruation and how to support females. Also, it could help to improve community attitudes towards menstruation.

## Supporting information

S1 File S1 GRAMMS checklist for the study. (DOCX)

S2 File S2 FGD guide use for the study. (DOCX)

S3 File S3 questionnaire use for the study. (DOCX)
