# Supplementary material for: Socio-cultural factors associated with knowledge, attitudes and menstrual hygiene practices among Junior High School adolescent girls in the Kpando district of Ghana: A mixed method study
Source: PLoS One. 2022 Oct 4;17(10):e0275583. doi: 10.1371/journal.pone.0275583 (PMC9531783; doi:10.1371/journal.pone.0275583)
Supplement: S1 File — (DOCX) [file pone.0275583.s001.docx]

**Good Reporting of A Mixed Methods Study (GRAMMS)**

| **Guideline** | **Section: page** |
| --- | --- |
| Describe the justification for using a mixed methods approach to the research question | Design: p8&9 |
| Describe the design in terms of the purpose, priority and sequence of methods | Design: p8&9 |
| Describe each method in terms of sampling, data collection and analysis | Data collection and analysis: p11-14 |
| Describe where integration has occurred, how it has occurred and who has participated in it | Design: p12&14 |
| Describe any limitation of one method associated with the present of the other method | Discussion: p30 |
| Describe any insights gained from mixing or integrating methods | Discussion: p26-30 |

*O'Cathain A, Murphy E, Nicholl J. The quality of mixed methods studies in health services research. J Health Serv Res Policy. 2008;13(2):92-98.*
