# Supplementary material for: Socio-cultural factors associated with knowledge, attitudes and menstrual hygiene practices among Junior High School adolescent girls in the Kpando district of Ghana: A mixed method study
Source: PLoS One. 2022 Oct 4;17(10):e0275583. doi: 10.1371/journal.pone.0275583 (PMC9531783; doi:10.1371/journal.pone.0275583)
Supplement: S2 File — (DOCX) [file pone.0275583.s002.docx]

**FGD GUIDE FOR FACILITATOR ON KNOWLEDGE ON MENSTRUATION AND SOCIOCULTURAL PRACTICES OF JUNIOR HIGH SCHOOL ADOLESCENTS IN THE KPANDO MUNICIPALITY**

**Introduction**

My name is ……… and I am conducting research on menstrual hygiene practices in your school.

I am much interested in your knowledge on menstruation as well as socio-cultural beliefs surrounding menstruation that is why I have invited you. So most of our conversation will focus on menstruation and socio-cultural beliefs surrounding menstruation and what your thoughts are about it. We really would love to know about what you think about menstruation. Also, this is a special gathering different from the classroom settings so there is no right or wrong answer. To this effect feel free to share your thoughts and opinions about menstruation. Every opinion will be accepted and appreciated so much.

Although we will listen to all your views, we may forget or will not be able to report exactly as you said so to that effect, _________ (name of note taker) will write down your opinions. However, she may not be able to capture all opinions in writing so we will also record the discussion so that we do not miss out on anything. I hope the recording is okay with you. (Wait for their response to confirm their consent before proceeding)

I also would like to assure you that every recorded information and the discussion as a whole will be kept with strictest confidentiality. In case you want to refer to a member of the group during the discussion, do not mention the name of the person, rather the number on the tags I have given you. So once again, do not be shy or scared to speak. Relax and voice out your opinions.

As much as we have gathered to have a discussion, we would have to adhere to some basic ground rules. These rules are just to keep the discussion organized. When a question is asked, all those who would like to say something about it would have to raise their hands and I will call you one after the other to give me your opinions. Do not be scared to disagree with opinions from colleagues. As much as possible, relax and share opinions.

***Instruction to moderator***

Before you begin, ask the students whether they have any questions before you begin? (if they have questions make sure you address them before you proceed)

*Rapport building*

Use the following questions as ice-breaker to make the students relax and do away with shyness.

As an introduction to the ice breakers, say let’s go around and have everyone tell us:

- Your favourite colour.
- Your favourite food and why do you love that particular food
- Where would you have chosen to be born if you had a choice and why?
- Your favourite subject in school and why?

During the introduction, look round to be sure all students are prepared, ready and are not showing any sign of shyness. Once you are sure that they are relaxed and not showing any sign of timidity or shyness, proceed to the actual discussion.

**Knowledge on menstruation**

1. What are some of the physiological changes girls experience as they grow?
2. Which health topics have you been taught in school?

Probe

- What were you taught under those topics? (probe for details about each topic respondents mentioned earlier)

1. Tell me what comes to your mind when you hear the word ‘menstruation’?
2. Who did you discuss your first menstruation with?

Probes

- Why did you discuss with that particular person and not any other person?
- What did he or she told you about menstruation?

1. Do you think menstruation is normal for adolescent girls and women? Why?
2. What are the community members’ perception about menstruation?

**Menstrual restrictions and related Socio cultural beliefs under pining these restrictions**

1. What are some of the restrictions on menstruating women in your community?

Probes

Are menstruating women allowed to;

- Cook for the family? Why?
- Bath with same bucket other use? Why
- Fetch water from the river? (supposing the community is along the river) Why
- Go to the farm (Why)
- What other things are you restricted from when you are menstruating?
- Share your personal experiences with these restrictions during menses?
- How do these restrictions make you feel?
- What are the consequences of violating theses norms?
- In your opinion, do you think these restrictions are fair to the women? Why?

**Thank you so much for your participation. We appreciate the time you spent with us and your opinions.**
