# Supplementary material for: Socio-cultural factors associated with knowledge, attitudes and menstrual hygiene practices among Junior High School adolescent girls in the Kpando district of Ghana: A mixed method study
Source: PLoS One. 2022 Oct 4;17(10):e0275583. doi: 10.1371/journal.pone.0275583 (PMC9531783; doi:10.1371/journal.pone.0275583)
Supplement: S3 File — (DOCX) [file pone.0275583.s003.docx]

**QUESTIONAIRE ON SOCIO-CULTURAL FACTORS ASSOCIATED WITH MENSTRUAL HYGIENE PRACTICES AMONG JUNIOR HIGH SCHOOL ADOLESCENTS IN THE KPANDO MUNICIPALITY**

This questionnaire seeks to collect information/data on demographics, knowledge on menstruation, and menstrual hygiene practices. Your name will not be written on the questionnaire to ensure anonymity. No answer provided will also be discussed with a third party and the completed questionnaire will be kept with outmost confidentiality. Also, you are expected to complete the questionnaire but if you decide otherwise, be assured that it would not affect your relationship with anyone in the school.

Kindly provide accurate answers by writing when required or tick appropriate answers where applicable

**SCHOOL NAME:**

**STUDENT NAME:**

**QUESTIONNAIRE NUMBER:**

**DATE:**

**Demographics**

1. Age_________
2. Age at first menses__________
3. Grade (1) JHS1 (2) JHS2 (3) JHS3
4. Religion (1) Christianity (2) Islamic (3) Traditional (4) Other ……..
5. Ethnicity (1) Ewe (2) Asante (3) Hausa (4)Ga (5) Other
6. Who do you live with? (1)Father (2) Mother (3)Both (4) Other Relatives (5) Other
7. Educational status of
   1. Mother (1) None (2) Primary (3)JHS (4) SHS (5)Tertiary
   2. Father (1) None (2) Primary (3)JHS (4) SHS (5)Tertiary
   3. Other Relatives (1) None (2) Primary (3)JHS (4) SHS (5)Tertiary
8. Guardian’s occupation (1) Father_____________ (2) Mother____________

(3) Other Relatives_____________ (4) Other

**Knowledge on menstruation**

1. What causes menstruation? (1) Hormones (2) Disease (3) Curses from gods (4) Don’t know (5) Other
2. Where does menstrual blood come from? (1)The womb/Uterus (2) Abdomen\stomach (3) Bladder (4) Vaginal (4) Don’t know
3. When a girl starts menstruating, what does it imply? (1) Capable of conceiving/entry into adulthood (2) Can start having sex intercourse (3) Ready for marriage (4) Others
4. What is the normal cycle length? (1) Less than 21days (2) 21-35days (3) More than 35days
5. Do boys menstruate? (1) Yes (2) No (3) Don’t know

**Hygienic practices**

1. What absorbent do you use during menstruation? (1) Sanitary pad (2) Toilet roll (3) Clean Cloth (4) Unclean Cloth (5) Other, specify_______
2. How many times do you change your absorbent daily? (1) Once (2) Twice (3) More than twice
3. Do you clean your genitalia daily during menstruation? (1) Yes (2) No

If yes, what do you clean your genitalia with during menstruation? (1) At least clean water (2) At least unclean water (3) Other

1. What sanitation facility do you use when you are menstruating? (1) Bathroom (2) Toilet (3) Urinal (4) Other, specify
2. Are there privacy options at where you change the absorbent? (1) Yes (2) No
3. What do you do with the absorbent after use? (1) Dispose of (2) Wash (3) Other, specify………(Skip Q23 & 24 if (1) applies or skip 21 & 22 if (2) applies)
4. Where do you dispose used absorbent? (1) Open field (2) Burn (3) Dustbin
   1. (4) Toilet (5) Others …………………
5. What material do you wrap the used absorbent in before disposing of? (1) Paper (2) Plastic (3)No wrapper (4) Other
6. What materials do you wash the absorbent with? (1) Water only (2) Water and soap (3) other, specify
7. Where do you dry the absorbent after washing? (1) Enclosed area without sunlight (2) Open place with sunlight

Thank you
